# Low central venous saturation predicts poor outcome in patients with brain injury after major trauma: a prospective observational study

**DOI:** 10.1186/1757-7241-17-23

**Published:** 2009-05-21

**Authors:** Alessandro Di Filippo, Chiara Gonnelli, Lucia Perretta, Giovanni Zagli, Rosario Spina, Marco Chiostri, Gian Franco Gensini, Adriano Peris

**Affiliations:** 1 Intensive Care Unit of Emergency Department, Careggi Teaching Hospital and University of Florence, Viale Morgagni 85, 50139, Florence, Italy; 2 Department of Critical Care Medicine and Surgery, University of Florence, Viale Morgagni 85, 50139, Florence, Italy

## Abstract

**Background:**

Continuous monitoring of central venous oxygen saturation (ScvO_2_) has been proposed as a prognostic indicator in several pathological conditions, including cardiac diseases, sepsis, trauma. To our knowledge, no studies have evaluated ScvO_2 _in polytraumatized patients with brain injury so far. Thus, the aim of the present study was to assess the prognostic role of ScvO_2 _monitoring during first 24 hours after trauma in this patients' population.

**Methods:**

This prospective, non-controlled study, carried out between April 2006 and March 2008, was performed in a higher level Trauma Center in Florence (Italy). In the study period, 121 patients affected by major brain injury after major trauma were recruited. Inclusion criteria were: 1. Glasgow Coma Scale (GCS) score ≤ 13; 2. an Injury Severity Score (ISS) ≥ 15. Exclusion criteria included: 1. pregnancy; 2. age < 14 years; 3. isolated head trauma; 4. death within the first 24 hours from the event; 5. the lack of ScvO_2 _monitoring within 2 hours from the trauma. Demographic and clinical data were collected, including Abbreviated Injury Scale (AIS), Injury Severity Score (ISS), Simplified Acute Physiologic Score II (SAPS II), Marshall score. The worst values of lactate and ScvO_2 _within the first 24 hours from trauma, ICU length of stay (LOS), and 28-day mortality were recorded.

**Results:**

Patients who deceased within 28 days showed higher age (53 ± 16.6 vs 43.8 ± 19.6, P = 0.043), ISS core (39.3 ± 14 vs 30.3 ± 10.1, P < 0.001), AIS score for head/neck (4.5 ± 0.7 vs 3.4 ± 1.2, P = 0.001), SAPS II score (51.3 ± 14.1 vs 42.5 ± 15, P = 0.014), Marshall Score (3.5 ± 0.7 vs 2.3 ± 0.7, P < 0.001) and arterial lactate concentration (3.3 ± 1.8 vs 6.7 ± 4.2, P < 0.001), than survived patients, whereas ScvO_2 _resulted significantly lower (66.7% ± 11.9 vs 70.1% ± 8.9 vs, respectively; P = 0.046). Patients with ScvO_2 _values ≤ 65% also showed higher 28-days mortality rate (31.3% vs 13.5%, P = 0.034), ICU LOS (28.5 ± 15.2 vs 16.6 ± 13.8, P < 0.001), and total hospital LOS (45.1 ± 20.8 vs 33.2 ± 24, P = 0.046) than patients with ScvO_2 _> 65%.

**Conclusion:**

ScvO_2 _value less than 65%, measured in the first 24 hours after admission in patients with major trauma and head injury, was associated with higher mortality and prolonged hospitalization.

## Background

Organ and tissue damages caused by a trauma impact lead to the development of systemic inflammatory response syndrome (SIRS) [1]. The local and systemic release of inflammation mediators produces oxidative stress, capillary leakage, microcirculatory disturbances, metabolic alteration, imbalance of pro- and anti-inflammatory mechanisms, ischaemia/reperfusion injury, coagulation disturbances [1]. In major trauma, occult tissue hypoperfusion within the first 24 hours after event precedes multiple organ dysfunction syndrome (MODS) [2]. Despite the unquestionable usefulness of routinely monitoring of hemodynamic parameters, such as arterial lactate, central venous pressure, blood pressure, heart rate, urinary volume, hypoxia may exist despite normal clinical values [3]. Continuous monitoring of central venous oxygen saturation (ScvO_2_) has been proposed as an indicator of tissue hypoperfusion. Although the reliability of ScvO_2_, compared with mixed venous oxygen saturation (SvO_2_), is still under discussion, particularly under shock conditions [3], the prognostic importance of low level (≤ 65%) of ScvO_2 _has been highlighted in severe sepsis [4,5], myocardial infarction [6], cardiac failure [7], and trauma [8]. Moreover, ScvO_2 _can be easily monitored with a central venous line, whereas SvO_2 _requires a pulmonary artery catheterization.

In a recent investigation about the cause of death in trauma Soreige K et al.[9] found a high percentage of head trauma (67%) and concluded that focus on brain injuries prevention is imperative.

To our knowledge, no studies have evaluated ScvO_2 _in patients affected by brain injury after major trauma so far. Thus, the aim of the present investigations was to assess the useful of an early monitoring (first 24 hours) of ScvO_2 _in patients with major trauma and head injury.

## Methods

### Data collection

The study population was recruited from the Intensive Care Unit (ICU) of Careggi Teaching Hospital (Florence, Italy) from 1st April 2006 to 31st March 2008. The Careggi Teaching Hospital is an a university-affiliated tertiary care hospital of Tuscany (IT) and acts as the provincial higher level trauma center for trauma. The ICU is a 10-single bed multidisciplinary medical/surgical/trauma unit that cares primarily patients of Emergency Department.

Patients were treated according to international standard care [10] and to Advanced Trauma Life Support guide lines (see Additional file 1), and were hemodynamically stabilized according to vital signs such as heart rate, blood pressure, and central venous pressure. Other parameters considered in the evaluation of severity of hypovolemia were the need for vasoactive drugs after hemodynamic resuscitation and the number of blood units administered. Inclusion criteria were: 1. admission diagnosis for major trauma with head injury; 2. a Glasgow Coma Scale (GCS) score ≤ 13, evaluated in the pre-hospital setting before sedation; 3. an Injury Severity Score (ISS) ≥ 15; 4. Exclusion criteria included: 1. pregnancy; 2. age < 14 years; 3. isolated head trauma; 4. death within the first 24 hours from the event; 5. the lack of ScvO_2 _monitoring within 2 hours from the trauma. This prospective observational study, followed the principles of the Helsinki declaration and national ethical guidelines. The study was authorized by the Local Scientific committee and the Local Ethic Committee, which waved the need of the informed consent because it did not modify the therapeutic approach provided by the current guide lines and internal protocols.

Patients' demographic and clinical characteristics were extracted from institutional ICU-database (software: FileMaker Pro 5.5v2, FileMaker, Inc, USA). All data have been collected prospectively by two trained data collectors. Patients has been anonymized, thus privacy was guarantee. Severity of disease was estimated by the Simplified Acute Physiology Score II (SAPS II) [11], a gravity score validated in 1993, submitted to a recent revision [12] (whose score is easily calculable on the site ), GCS, ISS, and AIS. The severity of the intracranial lesions was evaluated by the Marshall radiological score [13] (table 1) from the first CT-scan performed at hospital admission. Continuous monitoring of ScvO_2 _was performed with Vigileo system (Edwards Lifesciences, Irvine, California, USA). Central catheter was placed within the first two hours after admission in all patients enrolled. During the first 24 hours after trauma, both minimum value of ScvO_2_, measured for at least 15 minutes during the first 24 hours after trauma, and highest arterial lactate concentration were recorded. The data collectors verified that these values were not affected by episodic hypoxia during tracheal tube change, fibrobronchoscopy, occasional hypotension.

**Table 1:** Marshall Score [13].

	MARSHALL SCORE
Diffuse injury I	no visible pathology

Diffuse injury II	cisterns present, midline shift 0–5 mm and/or lesion densities present or no mass lesion > 25 ml

Diffuse injury III (swelling)	cisterns compressed or absent with midline shift 0–5 mm or no mass lesion > 25 ml

Diffuse injury IV (shift)	midline shift > 5 mm, no mass lesion > 25 ml; neurosurgery; high or mixed-density lesion > 25 ml, not surgically evacuated

The score is based on the severity of intracranial lesions identified at head CT scan

### Statistical Analysis

All analyses were performed using SPSS 10.0 statistical software package (SPSS, Chicago, Illinois, USA). Student's t-test was used for the numerical data, and two-tailed P value was considered significant if less than 0.05. Categorical data have been analysed with Fisher's exact test, with two-sided P significant if < 0.05. The cut-off values have been chosen based on statistically significant mean values of subgroups analysis. Multiple logistic regression has been performed on demographic and clinical parameters. A value of < 0.05 was considered significant.

## Results

### Overall population

In the study period, 121 patients met the inclusion criteria. 255 patients were admitted to the Emergency Room with ISS > 15 and GCS < 13; of these 21 died in the first 24 hours, 63 did not confirm a low GCS at the secondary evaluation and, in 50 of these the insertion of venous central catheter in the first 2 hours from trauma was not possible. Overall patients' demographic and clinical characteristics and comparison between 28-days survived and deceased patients are summarised in Table 2. Patients enrolled had a prevalence of head/neck and chest injury, as resulted from AIS score (Table 2). Male sex was predominant in both groups and represents 78% of overall population. This gender prevalence is in accordance on what usually observed in our clinical practice. On average, deceased patients showed a significant higher age (53 ± 16.6 vs 43.8 ± 19.6, P = 0.043), ISS core (39.3 ± 14 vs 30.3 ± 10.1, P < 0.001), AIS score for head/neck (4.5 ± 0.7 vs 3.4 ± 1.2, P = 0.001), SAPS II score (51.3 ± 14.1 vs 42.5 ± 15, P = 0.014), and Marshall Score (3.5 ± 0.7 vs 2.3 ± 0.7, P < 0.001), if compared with survived patients. Consistently with these observations, survived and deceased patients resulted significantly different in arterial lactate concentration (3.3 ± 1.8 vs 6.7 ± 4.2, respectively; P < 0.001) and ScvO_2 _(70.1% ± 8.9 vs 66.7% ± 11.9, respectively; P = 0.046) (Table 2).

**Table 2:** Demographic and clinical characteristics of overall population and comparison between survived and deceased patients at 28 days.

	Overall	Deceased	Survived	P
Number	121	22	99	
Age (years)	45.5 ± 19.3	53 ± 16.6	43.8 ± 19.6*	0.043
Male sex, % (N)	77.7% (94)	77.3% (17)	77.8% (77)	0.813
ISS score	32 ± 11.4	39.3 ± 14	30.3 ± 10.1*	< 0.001
AIS score	11.7 ± 3.3	12.6 ± 14.1	11.5 ± 3.1	0.117
*Head/Neck*	3.6 ± 1.2	4.5 ± 0.7	3.4 ± 1.2*	0.001
*Face*	1.8 ± 1.3	1.5 ± 1.2	1.8 ± 1.3	0.313
*Chest*	2.5 ± 1.1	2.8 ± 0.7	2.4 ± 1.2	0.495
*Abdominal*	1.0 ± 1.4	0.7 ± 1.1	1.1 ± 1.4	0.174
*Extremity*	1.5 ± 1.4	1.2 ± 1.6	1.6 ± 1.3	0.064
*External*	1.3 ± 0.8	1.3 ± 0.7	1.3 ± 0.8	0.886
SAPS II score	44.1 ± 15.2	51.3 ± 14.1	42.5 ± 15*	0.014
GCS score	7.2 ± 3.1	6.5 ± 3.2	7.4 ± 3	0.096
Marshall Score	2.6 ± 0.8	3.5 ± 0.7	2.3 ± 0.7*	< 0.001
ScvO_2_, %	70.9% ± 8	66.7% ± 11.9	70.1% ± 8.9*	0.046
HR (beats/min)	108 ± 23	110 ± 30	107 ± 28	0.744
MAP (mmHg)	79 ± 11	78 ± 16	80 ± 14	0.575
CVP (mmHg)	13 ± 2	12 ± 4	14 ± 6	0.861
Lactate (mmol/l)	4 ± 2.8	6.7 ± 4.2	3.3 ± 1.8*	< 0.001

ScvO_2 _level was the worst value measured for at least 15 minutes during the first 24 hours after trauma. Lactate concentration was the worst value measured during the first 24 hours after trauma.

Data are expressed as mean ± standard deviation (SD). Percent data are referred to the total population of each group.

Statistical analysis: two-tail Student's t-test. P-values were considered significant if less than 0.05 (*).

ISS: Injury Severity Score; AIS: Abbreviate Injury Scale; SAPS: Simplified Acute Physiology Score; GCS: Glasgow Coma Scale; ScvO_2_: central venous oxygen saturation; HR: heart rate; MAP: mean arterial pressure; CVP: central venous pressure.

### Mortality

The observed overall 28-days mortality rate was 18.2%. According with the differences between survived and deceased patients, contingency analysis showed that the significant relative risks for death were identified in age > 40 yrs, AIS (head/neck) ≥ 4, ISS score above 30, SAPS II score above 45, Marshall score > 2, lactate concentration > 3, and ScvO_2 _≤ 65% (Table 3). A multiple regression analysis performed on parameters with a higher correlation with 28-days mortality showed that variables which mostly contributed to the prediction of mortality were Marshall score (P = 0.001) and lactate concentration (P = 0.002). The calculation of concordance rate between two multiple regression analysis showed that adding ScvO_2 _to the other variables considered (age, AIS for head/neck, ISS, SAPS II, Marshall score and lactate concentration), raised the rate of correct predictions of 1% (from 86.6% to 87.6%).

**Table 3:** Results of the relative risk analysis (95% confidence interval) related to 28-day mortality.

	RR	95% CI	P	Sens	Spec	PPV	NPV
Age ≥ 40 *	2.7	1.1–7	0.032	0.77	0.46	0.25	0.91
Age ≥ 45	-	-	0.198	-	-	-	-
Age ≥ 50	-	-	0.113	-	-	-	-

AIS (*Head/Neck*) ≥ 3	-	-	0.272	-	-	-	-
AIS (*Head/Neck*) ≥ 4 *	4.1	1.3–13.1	0.007	0.86	0.46	0.26	0.94

ISS ≥ 30 *	3	1.2–7.7	0.017	0.77	0.53	0.27	0.91
ISS ≥ 35 *	3.8	1.7–8.7	0.001	0.68	0.71	0.35	0.91
ISS ≥ 40 *	2.4	1.149–5	0.03	0.46	0.79	0.32	0.87

SAPS II ≥ 40	-	-	0.188	-	-	-	-
SAPS II ≥ 45 *	2.9	1.2–6.9	0.017	0.73	0.58	0.28	0.91
SAPS II ≥ 50 *	2.3	1.1–4.8	0.046	0.55	0.7	0.29	0.87

Marshall score ≥ 2 *	14.7	3.6–60.1	< 0.001	0.91	0.71	0.41	0.97
Marshall score ≥ 3 *	5.8	2.8–12	< 0.001	0.59	0.89	0.54	0.91

Lactate ≥ 3 mmol/l *	6.8	1.7–27.8	< 0.001	0.90	0.51	0.30	0.96
Lactate ≥ 4 mmol/l *	4.8	1.9–12.3	< 0.001	0.75	0.70	0.37	0.92
Lactate ≥ 5 mmol/l *	4.2	2–9	< 0.001	0.55	0.85	0.46	0.89

ScvO_2 _≤ 65% *	2.3	1.1–4.8	0.034	0.46	0.78	0.31	0.87
ScvO_2 _≤ 70%	-	-	0.186	-	-	-	-

The cut-off values have been chosen based on statistically significant mean values of subgroups analysis (survived vs deceased patients, see Table 2).

Statistical analyses were performed using the two-tails Fisher's exact test (95% confidence interval). P-values were considered significant if less than 0.05 (*).

RR: Relative Risk; CI: Confidence Interval; Sens: Sensitivity; Spec: Specificity; PPV = Positive Predictive Value; NPV = Negative Predictive Value; SAPS: Simplified Acute Physiology Score; GCS: Glasgow Coma Scale; ISS: Injury Severity Score; AIS: Abbreviate Injury Scale; ScvO_2_: central venous oxygen saturation.

### Subgroup analysis

Subgroup analysis of overall population, based on an ScvO_2 _value of ≤ 65%, showed that patients with worst ScvO_2 _had a significantly higher 28-days mortality rate (31.3% vs 13.5%, P = 0.034) respect to the comparison group (Table 4). Also total intra-ICU mortality (34.4% vs 13.5%, P = 0.017) and total intra-hospital mortality (37.5% vs 14.6%, P = 0.010) resulted significantly higher in ScvO_2 _≤ 65% group respect to the ScvO_2 _> 65% group (Table 4). Moreover, the ScvO_2 _≤ 65% group significantly differed in age (54.5 ± 18.3 vs 42.2 ± 18.8, P = 0.002), ISS score (36.1 ± 10.3 vs 30.5 ± 11.5, P = 0.018), total AIS score (12.94 ± 2.9 vs 11.3 ± 3.3, P = 0.016), AIS score for extremity (2.2 ± 1.7 vs 1.3 ± 1.2, P = 0.001), SAPS II score (51.1 ± 15.0 vs 41.6 ± 14.5, P = 0.002), and GCS score (5.7 ± 2.7 vs 7.8 ± 3.0, P = 0.001) from patients with ScvO_2 _> 65% (Table 4). Conversely, Marshall score and lactate values did not differ significantly between the two groups (Table 4).

**Table 4:** Comparison of demographic and clinical characteristics of overall patients.

	Overall patients		
	
	ScvO_2 _≤ 65%	ScvO_2 _> 65%	P
Number	32	89	0.002
Age (years)	54.5 ± 18.3	42.2 ± 18.8*	0.491
Male sex, % (N)	71.9% (23)	79.8% (71)	0.018
ISS score	36.1 ± 10.3	30.5 ± 11.5*	0.016
AIS score§	13 ± 11,5	10 ± 9*	0.324
*Head/Neck*	3 ± 1	3 ± 1	0.674
*Face*	2 ± 1	2 ± 1	0.625
*Chest*	3 ± 1	2.5 ± 1	0.38
*Abdominal*	1 ± 1	1 ± 1	< 0.001
*Extremity*	2 ± 1	1 ± 1*	0.362
*External*	1 ± 1	1 ± 1	0.002
SAPS II score	51.1 ± 15.0	41.6 ± 14.5*	0.001
GCS score	5.7 ± 2.7	7.8 ± 3.0*	0.109
Marshall Score§	2.5 ± 2	2 ± 2	< 0.001
ScvO_2_. %	59% ± 5	74% ± 6*	0.078
Lactate (mmol/l)	4.5 ± 2.4	3.7 ± 2.9	0.127
HR (beats/min)	113 ± 21	109 ± 27	0.211
MAP (mmHg)	78 ± 14	82 ± 13	0.344
CVP (mmHg)	11 ± 3	11 ± 2	< 0.001
ICU LOS (days)	28.5 ± 15.2	16.6 ± 13.8*	0.046
Hospital LOS (days)	45.1 ± 20.8	33.2 ± 24*	0.034
28-days mortality, % (N)	31.3% (10)	13.5% (12)*	0.017
Total intra-ICU mortality, % (N)	34.4% (11)	13.5% (12)*	0.010
Total intra-hospital mortality, % (N)	37.5% (12)	14.6% (13)*	0.002

Subgroups analysis was based on the ScvO_2 _values (≤ 65% vs > 65%) measured during the first 24 hours from trauma event. ScvO_2 _level was the worst value measured for at least 15 minutes during the first 24 hours after trauma. Lactate concentration was the worst value measured during the first 24 hours after trauma. ICU LOS and hospital LOS have been calculated on patients discharged alive from ICU and from hospital, respectively. Total intra-ICU mortality included one patient died in ICU after readmission.

Data are expressed as mean ± standard deviation (SD). Percent data are referred to the total population of each group.

Statistical analysis: two-tails Student's t-test, two-sides Fisher's exact test. P-values were considered significant if less than 0.05 (*).

ISS: Injury Severity Score; AIS: Abbreviate Injury Scale; SAPS: Simplified Acute Physiology Score; GCS: Glasgow Coma Scale; ScvO_2_: central venous oxygen saturation; HR: heart rate; MAP: mean arterial pressure; CVP: central venous pressure.

§ Median ± interquartile range

## Discussion

The aim of the present investigation was to assess the role of early ScvO_2 _monitoring in trauma patients with associated head injury. We assumed that a persistent low ScvO_2 _could be the expression of the dangerous effects of generalized and, concomitant, brain hypoperfusion in patients with brain injury and major trauma and, therefore, that it could predict a worst evolution.

The prognostic importance of low level (≤ 65%) of ScvO_2 _has been emphasized in severe sepsis [4,5], myocardial infarction [6], and cardiac failure [7]. The significance of ScvO_2 _in patients with major trauma has been previously reported in 26 injured patients [8]. In their investigation, Scalea and co-workers found that ScvO_2 _measurement was a trustworthy parameter to estimate blood loss, especially in patients who showed an ScvO_2 _below 65% despite stable clinical signs [8]. In opposition with this observation, a subsequent study did not confirm the reliability of ScvO_2 _as indicator of blood loss in 40 trauma patients [14]. According with Scalea *et al*., the lack of linear correlation between low ScvO_2 _and stable clinical signs has been also corroborated by Rady and colleagues in a nonrandomized study on sixteen patients presenting to the emergency department in shock conditions, who presented ScvO_2 _below 65% in almost 50% of cases, despite the hemodynamic stabilization [5].

The ScvO_2 _cut-off value of ≤ 65% has been chosen in the present investigation based on existing literature [5,8,14] and on the average we found in our patients deceased at 28 days (66.7%, Table 2). The main finding of this study was the relationship between an ScvO_2 _values ≤ 65%, for at least 15 minutes during the first 24 hours from trauma, and mortality rates (Table 4). It must be noted that in the group with lower ScvO_2 _also injury scores and lactate levels were worst. In regard, the absolute significance of ScvO_2 _in predicting mortality in the multivariate analysis was lower than Marshall score and lactate levels in our investigation. However, the inclusion of ScvO_2 _in a multiple regression model showed that mortality prediction can be improved from 86.6% to 87.6%: this improvement, whereas not ample, could be relevant in the day to day clinical practice. In consideration of our results, ScvO_2_should be never considered as a substitute of lactate monitoring or injury scores in predicting outcome. In addition to these observations, patients with ScvO_2 _values > 65% discharged alive from ICU, showed significantly reduction in both ICU and total hospital LOS (Table 4). Our findings are partially in accordance with a recent study performed on 96 critically ill patients admitted in a multidisciplinary ICU, in which an ScvO_2 _value below 60% at admission was related with high mortality rate but not with a prolonged ICU LOS [15]. On regard, it must be considered that the study of Bracht and co-workers was performed on unselected critically ill patients, with a not specified number of central nervous system injured patients (less than 30% of total patients enrolled), whereas our population included polytraumatized patients with head injury. In our study, the association between low level of ScvO_2 _and ICU/hospital LOS appeared strong; however, this finding could be also related with the differences in age and injuries observed in the two subgroups (Table 4).

The most numerous and extensive studies on ScvO_2 _optimization have been performed on septic patients. To our knowledge, no study has investigated the usefulness of ScvO_2 _monitoring to guide hemodynamic stabilization and to improve outcome in patients with major trauma [3]. The pathogenic changes following major trauma include SIRS, oxidative stress, capillary leakage, metabolic alterations, and diffuse tissue hypoxia as a result of circulatory abnormalities [1]. This pathophysiological process is somehow very similar with what observed in septic patients, although sepsis presents a different aetiology, evolution and possibility to be treated with antibiosis. In our investigation, ScvO_2 _was not included as a target parameter for hemodynamic optimization, so none can be gathered from our data on application of early-goal directed therapy protocol in major trauma. Nevertheless, considering the good evidence in patients with severe sepsis/septic shock, the use of ScvO_2 _to guide hemodynamic optimization in polytraumatized patients would merit to be adequately investigated.

Limits of present findings merit considerations. 1) We did not included data on fluids administration in the first 24 hours, brain damages evolutions, intracranial pressure, and their relationship with ScvO_2 _levels. Second, the exclusion of patients who died in the first 24 hours from the study protocol might be a potential bias. The reason of this choice depended from our goal to investigate the role of ScvO_2 _in the first 24 hours, which are considered critical in the evolution of MODS in polytrauma [2]. Third, we were not blinded for the primary end points (but in an emergency setting is not so easy), but the collection of data was made by an external investigator that was blind for the end points. Finally, the lack of a control group with a pulmonary artery catheter with SvO_2 _monitoring did not allow a comparison between ScvO_2 _and SvO_2_, although previous studies showed that changes of both values proceed in a parallel manner, even under shock conditions [3,16].

## Conclusion

In conclusion, an early monitoring of ScvO_2 _could aid to predict the outcome in patients with major trauma and head injury. In particular, we found that a value less than 65%, measured during the first 24 hours after admission, was associated with higher mortality rate and prolonged ICU and hospital LOS. So, our results seem to suggest the use of ScvO_2 _as a target of resuscitation in patients with brain injury after major trauma. The validation of ScvO_2 _in monitoring of cerebral metabolism and as a target for hemodynamic resuscitation in polytraumatized patients remains to be investigated.

## Key messages

• ScvO_2 _should be monitored in patients with major trauma, especially if head injury coexists.

• ScvO_2 _value less than 65%, measured in the first 24 hours after admission, is associated with higher mortality and prolonged ICU/hospital LOS.

• ScvO_2 _as a prognostic indicator should be considered in its relationship with typology of injuries and the other illness scores.

## Abbreviations

AIS: abbreviated injury scale; GCS: Glasgow coma scale; ICU: intensive care unit; ISS: injuryseverity score; LOS: length of stay, MODS: multiple organ dysfunction syndrome; SAPS II: simplified acute physiology score II; SIRS: systemic inflammatory response syndrome; ScvO_2_: central venous oxygen saturation; SvO_2_: mixed venous oxygen saturation.

## Competing interests

The authors declare that they have no competing interests.

## Authors' contributions

ADF and AP planned the study and reviewed the literature. ADF, CG and AP wrote the manuscript. MC and GZ performed the statistical analyses. GFG, RS, LP and CG participated in the design ofthe study. LP and CG collected data and helped draft the manuscript. GZ revised the manuscript. All authors have seen and approved the final revised version.

## Supplementary Material

Additional file 1 **Appendix on trauma**. Major diagnostic and therapeutic procedures in the treatment of trauma in the Tuscany Region, Italy. Click here for file
