# Supplementary material for: Low central venous saturation predicts poor outcome in patients with brain injury after major trauma: a prospective observational study
Source: Scand J Trauma Resusc Emerg Med. 2009 May 21;17:23. doi: 10.1186/1757-7241-17-23 (PMC2694764; doi:10.1186/1757-7241-17-23)
Supplement: Additional file 1 — Appendix on trauma. Major diagnostic and therapeutic procedures in the treatment of trauma in the Tuscany Region, Italy. [file 1757-7241-17-23-S1.doc]

|  | ***Diagnostic*** | ***Procedure/Therapeutic*** | *Notes* |
| --- | --- | --- | --- |
| **ON THE FIELD**  Helicopter service (anaesthetist)  ALS medical car  BLSD ambulance | Clinical examination  basic monitoring | ATLS  Primary survey  Secondary survey | Centralization on higher level trauma center (in Tuscany: University Hospital of Pisa, Siena and Firenze) |
| **EMERGENCY ROOM**  Emergency physician  Emergency Nurses  Anaesthetist  Radiologist  Emergency Surgeon  and sub-speciality | FAST  Central venous catheter insertion (ScvO_2_, Cardiac Index, Central Venous Pressure)  Total Body CT scan  Angiography | Airway management  Ventilation  Fluids administration targeted on ScvO_2_  Fibrinogen, 0-NEG Blood Units, Fresh Frozen Plasma, Coagulation Factors, aFactorVII  Emergency surgery and sub-speciality: Damage control surgery  Embolization  Early neuro-protection | Periodic revision and updating of Guidelines |
| **ICU**  Anaesthetist  ICU Nurses | Intracranial pressure monitoring  Brain tissue oxygen tension  SjO_2_  EEG and SSEP  Haemodynamic advanced monitoring  ScvO_2_  Bedside Thorax, Vascular, Cardiac trans-cranial doppler Echography  Trans-oesophageal echocardiography  CT scan and X ray | Cerebral pressure perfusion control  Sedation  Effects of ventilation management  Decompressive craniotomy  Early tracheostomy  Protective ventilation  Nitric Oxyde administration  Extracorporeal Membrane Oxygenation  Echo guided drainage of collections  Haemodynamic optimization  CVVH  Diagnostic bedside Video-laparoscopy  Surgical control of Abdominal Compartment Syndrome | Periodic revision and updating of Guidelines  Antibiotic policy and infection control programme  Early physiotherapy  Trauma registry |

Appendix 1: Major diagnostic and therapeutic procedures in the treatment of Trauma in the Tuscany Region, Italy. (For further information please contact the authors)

ALS: Advanced Life Support; BLSD: Basic Life Support and Defibrillation; ATLS: Advanced Trauma Life Support; FAST: Focused Abdominal ultraSound Trauma; ScvO_2_: Saturation central venous O_2_; CT: Computerized Tomography; ICU: Intensive Care Unit; SjO_2_: Saturation jugular O_2_; EEG: Electroencephalography; SSEP: Somato-Sensorial Evoked Potentials; CVVH: Continuous veno-venous Hemofiltration
